# Brassinosteroids as phytohormonal shields against micro- and nanoplastic stress in plants

**DOI:** 10.3389/fpls.2026.1920456

**Published:** 2026-07-21

**Authors:** Andrzej Bajguz, Jan Żeruń

**Affiliations:** 1 Department of Biology and Plant Ecology, Faculty of Biology, University of Bialystok, Bialystok, Poland; 2 Doctoral School of University of Bialystok, Bialystok, Poland

**Keywords:** AsA-GSH cycle, brassinosteroids, microplastics, nanoplastics, oxidative stress, rhizosphere microbiome

## Abstract

Microplastics (MPs) and nanoplastics (NPs) are biologically active stressors in agricultural soils, where they alter soil physical structure, disrupt rhizosphere processes, impair water and nutrient acquisition, and provoke oxidative and hormonal disequilibrium in plants. Brassinosteroids (BRs), particularly brassinolide and 24-epibrassinolide, have recently emerged as modulators of plant responses to plastic-particle stress. Current evidence indicates that BRs do not detoxify plastics directly. Instead, they reorganize plant performance across interconnected layers: aquaporin-linked NP transport, antioxidant and ascorbate-glutathione metabolism, photosystem II function, hormone crosstalk, secondary metabolism, and rhizosphere feedbacks. In tomato, BRs reduced polystyrene-NP accumulation in edible tissues by suppressing aquaporin genes. In *Pinellia ternata* and rice, BRs attenuated MP/NP-induced growth inhibition by restoring photosynthetic efficiency and redox control. This mini review synthesizes these findings and frames BRs as eco-hormonal regulators of the plant-plastic-soil interface, while highlighting priorities for field-realistic validation.

## 1 Introduction

Plastic particles have become persistent constituents of agricultural systems. Microplastics (MPs), generally defined as particles <5 mm, and nanoplastics (NPs), <1 µm, originate from plastic mulch residues, irrigation water, sewage sludge, compost, agrochemical packaging, and atmospheric deposition (Jia et al., 2023; Arif et al., 2024; Saeed et al., 2026). However, they are not inert once in soil. Their polymer chemistry, size, shape, charge, aging state, and biofilm or co-contaminant load determine mobility, contact with roots, and biological effects. Larger MPs mainly act through soil-mediated mechanisms, including altered aggregation, porosity, water retention, porewater conductivity, and microbial activity (Qiu et al., 2022), whereas smaller particles and NPs interact more directly with root surfaces and, under specific experimental conditions, may be internalized and transported to shoots or edible organs (Taylor et al., 2020; Spanò et al., 2022; Zytowski et al., 2025).

At the plant level, MP/NP stress manifests as a syndrome characterized by inhibited germination or root elongation, altered nutrient and water uptake, reactive oxygen species (ROS) overproduction, lipid peroxidation, reduced chlorophyll content, impaired photosystem II (PSII) efficiency, hormonal disruption, and carbon reallocation toward defense mechanisms (Lima et al., 2023; Zhuang et al., 2025). Severity is strongly context-dependent. Particle size and dose are critical, as shown in rice seeds where 50 nm polystyrene particles were more inhibitory than 50 µm particles (Gui et al., 2025). Plastic type and co-occurring plasticizers also reshape hormonal and metabolic networks, as shown in cucumber exposed to polyethylene, polystyrene, polyvinyl chloride, and di-*n*-octyl phthalate (Zhuang et al., 2025). Therefore, mitigation should target not only plastic removal but also the biochemical state generated by chronic plastic exposure.

Brassinosteroids (BRs) are steroidal phytohormones controlling cell expansion, vascular development, photosynthesis, source-sink relations, antioxidant defense, and hormonal crosstalk. BRs commonly enhance antioxidant capacity, stabilize chloroplast function, sustain carbon assimilation, and buffer stress-induced shifts in abscisic acid (ABA), auxin, cytokinin (CK), gibberellin (GA), ethylene (ET), jasmonate (JA), and salicylate pathways, thereby helping plants maintain growth while avoiding excessive stress signaling. Beyond their developmental role, BRs are widely recognized as broad-spectrum modulators of plant acclimation to abiotic constraints, including drought, salinity, temperature extremes, heavy-metal toxicity, nutrient imbalance, and oxidative stress. In drought- or salinity-exposed plants, BRs commonly improve water relations, sustain membrane stability, regulate osmotic adjustment, and strengthen antioxidant protection. Under temperature stress, they contribute to the preservation of chloroplast function, photosystem II efficiency, and carbon assimilation. In metal- or nutrient-related stress, BRs can modulate ion homeostasis, redox metabolism, and stress-responsive hormonal networks. These examples are relevant to MP/NP stress because plastic particles do not impose a single isolated injury. Instead, they generate a composite stress syndrome involving impaired water and nutrient acquisition, oxidative damage, photosynthetic inhibition, hormonal imbalance, altered root architecture, and rhizosphere disturbance (Ahanger et al., 2018; Planas-Riverola et al., 2019; Nolan et al., 2020; Chakraborty et al., 2025). Therefore, the well-established capacity of BRs to coordinate redox control, photosynthetic performance, osmotic balance, growth-defense trade-offs, and hormone crosstalk provides a mechanistic rationale for considering BRs as plausible candidates for MP/NP stress mitigation. However, direct evidence connecting canonical BRI1-BIN2-BZR1 signaling modules with MP/NP-specific responses remains scarce. Thus, the proposed role of BR signaling in plastic-particle stress should be regarded as a mechanistic hypothesis extrapolated from better-characterized abiotic-stress models and requiring validation in BR-deficient, BR-insensitive, and BR-overaccumulating genotypes.

## 2 Brassinosteroid responses to MP/NP stress

Direct evidence that BRs help mitigate MP/NP stress in plants is still emerging, but the available studies suggest a coherent regulatory framework. Plastic particles impose a composite abiotic stress involving physical interference at the root interface, oxidative injury, impaired photosynthesis, hormonal imbalance, metabolic reallocation, and rhizosphere disruption. BRs appear to counter this syndrome by acting across the same organizational levels. The following sections therefore examine BR action from particle exclusion and redox repair to photosynthetic recovery, hormone-metabolite reprogramming, and plant-soil feedback stabilization.

### 2.1 Direct evidence for BR-mediated mitigation of plastic-particle stress

Although direct evidence remains limited, available studies converge on three distinct mechanistic levels of BR action: reduced plastic-particle accumulation, restoration of plant physiological homeostasis, and stabilization of the plant-soil interface. In tomato plants exposed to polystyrene NPs, exogenous BRs reduced NP accumulation in fruit and mitigated toxicity by activating antioxidant defenses and suppressing NP-induced aquaporin genes, including TIP2-1, PIP2-6, PIP2-8, and NIP1-2 (Gao et al., 2023). In *Pinellia ternata* cultivated in the presence of 1% polyethylene MPs, foliar application of brassinolide (BL) counteracted growth inhibition and restored physiological performance, increasing plant height, aboveground and underground biomass, carotenoid and glutathione levels, Fv/Fm, and the activities of superoxide dismutase (SOD), glutathione reductase (GR), and monodehydroascorbate reductase (MDHAR) under MP stress (Zhang et al., 2024). In rice seeds exposed to 50 µm and 50 nm polystyrene particles, 24-epibrassinolide (24-epiBL) improved antioxidant capacity and alleviated growth inhibition, with the protective effect being particularly pronounced under nanoplastic exposure (Gui et al., 2025).

A complementary study in *P. ternata* extended BL action beyond plant tissues to the rhizosphere, showing that foliar BL modulated soil enzyme activities and microbial community composition under MP contamination (Zhang et al., 2025). Collectively, these studies support a working model in which BRs alleviate plastic-particle stress through pollutant-exclusion mechanisms, biochemical repair, and plant-soil stabilization, rather than via direct plastic degradation.

### 2.2 Limiting nanoplastic uptake: aquaporins and the root interface

Particle uptake remains a central unresolved issue in the study of plant-plastic interactions. MPs can adhere to the root cap and root hairs, block seed-coat or root pores, and modify rhizosphere hydraulics. NPs, because of their high surface area and small size, are more likely to interact with cell walls, apoplastic spaces, endocytic routes, and membrane-associated transport processes (Taylor et al., 2020; Spanò et al., 2022; Zytowski et al., 2025). The tomato study is particularly noteworthy, as BL treatment suppressed PS-NP accumulation in edible tissues and downregulated several aquaporin genes (Gao et al., 2023). Aquaporins are primarily water and small-solute channels (Maurel et al., 2008; Tyerman et al., 2021); therefore, these data should not be interpreted as proof that NPs pass directly through aquaporin pores. A more defensible interpretation is that BRs reduce the hydraulic and membrane-transport permissiveness associated with NP translocation by lowering aquaporin-linked water flux, stabilizing membranes, and reconfiguring root-to-shoot transport. This mechanism is likely particle-size dependent: for larger MPs, protection may depend more on improved root vigor, cell-wall integrity, osmotic adjustment, and soil-root interface function. An additional layer may involve BR-dependent modification of the root apoplast. By influencing cell-wall remodeling, membrane stability, and possibly lignin or suberin deposition, BRs could alter the physical permeability of root tissues to particle-associated stress. For NPs, BR regulation of PIP, TIP, and NIP aquaporins offers a testable route for limiting xylem loading and edible-tissue accumulation (Gao et al., 2023).

### 2.3 Redox control and the AsA-GSH cycle

Oxidative stress is the most consistent biochemical signature of MP/NP exposure. Plastic particles can elevate the levels of superoxide, hydrogen peroxide, and malondialdehyde while disrupting antioxidant enzymes activities in a species-, tissue-, and dose-dependent manner (Jia et al., 2023; Zhang et al., 2024; Gui et al., 2025). BRs appear to shift plants from uncontrolled ROS accumulation to regulated redox homeodynamics. In *P. ternata*, MP exposure reduced SOD and ascorbate peroxidase (APX) activities but increased superoxide production; under MP stress, BR application enhanced SOD, GR, and MDHAR activities and glutathione levels, indicating reinforcement of the ascorbate-glutathione (AsA-GSH) cycle (Zhang et al., 2024). In rice seeds, improved growth under BL supplementation under plastic stress was associated with stronger antioxidant capacity (Gui et al., 2025).

At the biochemical level, this protective effect is consistent with BR-dependent reinforcement of the AsA-GSH redox hub. SOD converts superoxide to hydrogen peroxide; APX, catalase (CAT), and peroxidases detoxify hydrogen peroxide; GR regenerates reduced glutathione; and MDHAR/dehydroascorbate reductase maintain the ascorbate pool (Foyer and Kunert, 2024). By preserving this redox network, BRs protect chloroplast membranes, proteins, and nucleic acids while maintaining ROS as signaling molecules required for acclimation. Thus, BRs likely restore ROS homeostasis rather than simply suppressing ROS (Gui et al., 2025; Zhang et al., 2025).

### 2.4 Photosynthetic rescue and source-sink stabilization

Photosynthesis is highly sensitive to plastic-induced stress. MPs/NPs can reduce chlorophyll biosynthesis, destabilize thylakoid membranes, impair electron transport, and decrease PSII efficiency, thereby restricting carbon assimilation (Jia et al., 2023; Zhang et al., 2024). BRs counteract this vulnerability at several levels. In *P. ternata*, MP exposure reduced Fv/Fm, whereas BR supplementation increased both Fv/Fm and carotenoid content (Zhang et al., 2024). Carotenoids quench triplet chlorophyll and singlet oxygen, making their recovery directly relevant to photoprotection. In tomato exposed to PS-NPs, BL also improved photosynthetic parameters and Rubisco-associated performance, linking redox repair to carbon metabolism (Gao et al., 2023). The growth recovery observed after BR treatment should therefore be viewed as coordinated source-sink stabilization. By maintaining PSII efficiency, antioxidant protection, and hormone balance, BRs preserve carbon gain while limiting excessive defense costs. However, BR responses are dose- and organ-dependent. In the *P. ternata* rhizosphere study, BL alone reduced root length, and the combined BL+MP treatment further reduced it (Zhang et al., 2025). A shorter but more protected root system may be adaptive under stress, but this requires anatomical and hydraulic validation.

### 2.5 Hormonal and metabolic reprogramming

Plastic-induced stress disrupts the hormonal network that balances growth with defense (Jia et al., 2023; Zhang et al., 2025). Multiomics analysis in cucumber showed that MPs and di-*n*-octyl phthalate affected tryptophan-dependent auxin biosynthesis, CK *de novo* synthesis, GA metabolism, ABA-related ABA2 expression, ethylene biosynthesis, jasmonic acid accumulation, and endogenous BR homeostasis through CYP734A1-linked regulation (Zhuang et al., 2025). BRs are well positioned to rectify such imbalance because BR signaling intersects with auxin transport and sensitivity, GA-DELLA control, ABA-mediated stress responses, ethylene, jasmonate, and salicylate pathways (Ahanger et al., 2018; Planas-Riverola et al., 2019; Nolan et al., 2020).

Metabolically, BRs influence osmoprotectants, amino acids, sugars, fatty acids, and secondary metabolites. In *P. ternata*, MP exposure enhanced flavonoids, alkaloids, and β-sitosterol while suppressing growth, suggesting a reallocation of carbon resources toward defense and medicinal secondary metabolism (Zhang et al., 2024). BL treatment restored growth and redox balance, implying partial reallocation from emergency defense toward productive acclimation. This is important for edible and medicinal plants, where mitigation should preserve biomass, safety, and metabolite quality.

### 2.6 Rhizosphere stabilization and plant-soil feedbacks

MP toxicity is not restricted to plant tissues. MPs alter soil moisture, pH, electrical conductivity, nutrient availability, enzyme activities, aggregate structure, and microbial networks (Lima et al., 2023; Zhang et al., 2025; Saeed et al., 2026). Because BRs are usually applied to shoots, their rhizosphere effects are likely mediated through altered root growth, exudation, nutrient demand, and stress signaling. In *P. ternata*, foliar BL under MP stress decreased soil catalase activity but increased urease activity, suggesting alteration in microbial metabolic function and nitrogen cycling. BL also partially mitigated MP-induced microbial dysbiosis, including shifts in dominant bacterial groups such as Proteobacteria and Actinobacteriota and changes in fungal taxa including Plectosphaerella, Fusarium, and Rhizoctonia (Zhang et al., 2025). These findings expand the BR concept from a plant-internal stress hormone to an eco-hormonal regulator of the plant-plastic-soil interface. The exact causal pathways remain to be elucidated and warrant further investigation using root exudomics, metatranscriptomics, enzyme assays, and microbial isolation experiments.

## 3 Future directions and conclusion

**Figure 1:**
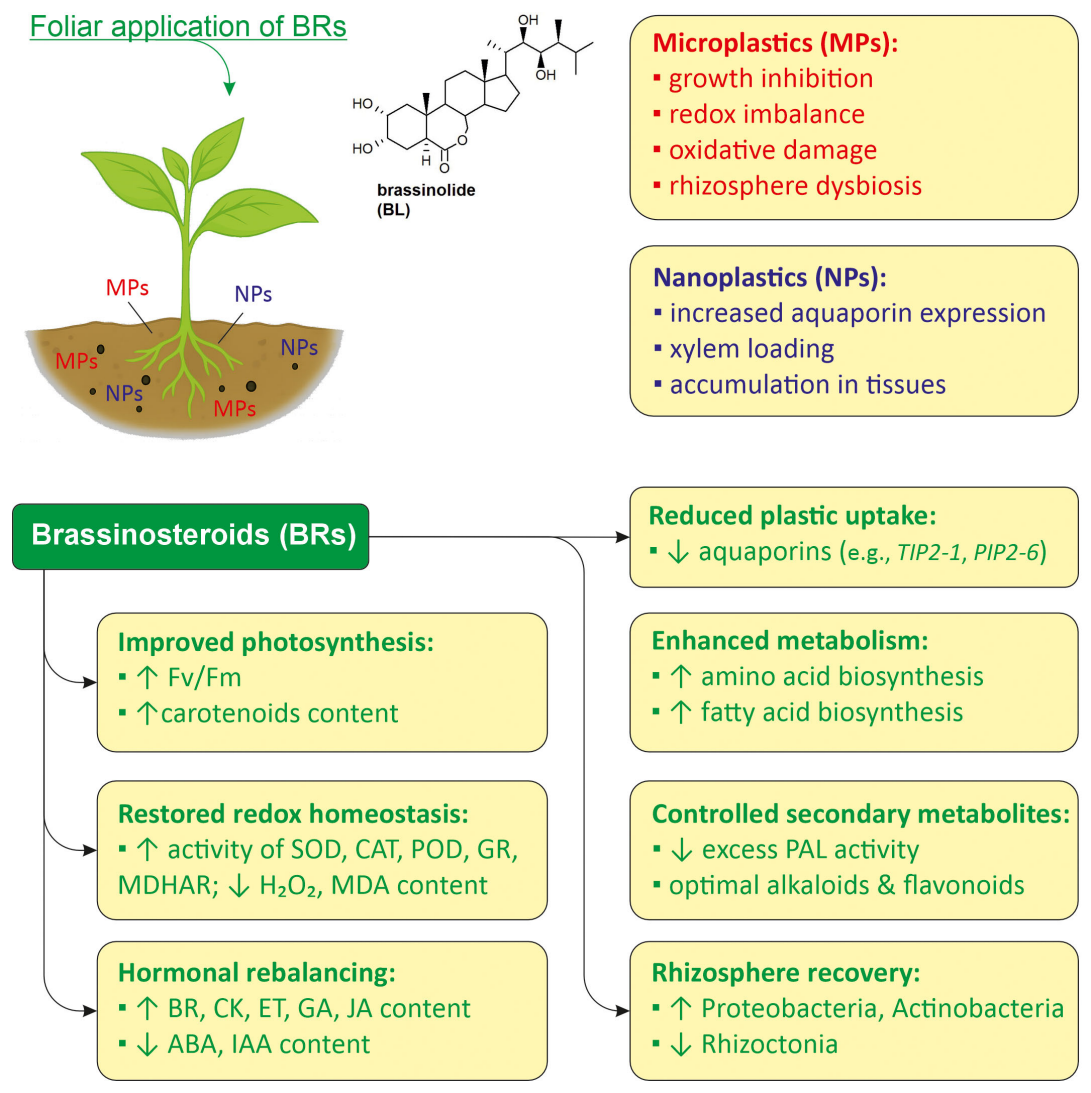
BR-mediated reprogramming of plant responses to MP and NP stress. Plastic particles impair growth by disturbing redox homeostasis, photosynthetic efficiency, hormonal balance, metabolism, and rhizosphere stability. NPs may additionally enhance aquaporin-associated uptake, xylem loading, and tissue accumulation. Foliar BRs, particularly BL, mitigate plastic-induced phytotoxicity by downregulating selected aquaporins, enhancing antioxidant defenses, restoring PSII performance, rebalancing endogenous phytohormones, sustaining metabolism, and partially recovering rhizosphere microbial structure. ABA, abscisic acid; APX, ascorbate peroxidase; BL, brassinolide; BR, brassinosteroid; CAT, catalase; CK, cytokinin; ET, ethylene; GA, gibberellin; GR, glutathione reductase; H_2_O_2_, hydrogen peroxide; IAA, indole-3-acetic acid; JA, jasmonate; MDA, malondialdehyde; MDHAR, monodehydroascorbate reductase; MP, microplastic; NP, nanoplastic; PAL, phenylalanine ammonia-lyase; POD, peroxidase; SOD, superoxide dismutase; Fv/Fm, maximum quantum efficiency of PSII.

Current evidence indicates that BRs mitigate MP/NP phytotoxicity through integrated regulation of transport, redox homeostasis, photosynthesis, hormone signaling, metabolism, and rhizosphere function (**Figure 1**; **Table 1**). Prior to successful agronomic translation, four limitations require attention. First, most studies use pristine polystyrene or polyethylene particles at relatively high concentrations, whereas field soils contain aged, irregular, biofilm-coated plastics contaminated with additives, pesticides, and metals (Lima et al., 2023; Arif et al., 2024; Saeed et al., 2026). Second, BR dose, timing, formulation, and application route must be optimized because excessive BR can disturb root growth or hormonal balance. Third, mechanisms should be validated genetically using BR signaling mutants, aquaporin mutants, and inhibitors rather than physiological endpoints alone (Nolan et al., 2020; Tyerman et al., 2021). Fourth, future experiments must quantify edible-tissue plastic accumulation, yield, nutritional quality, and secondary metabolites over complete crop cycles. Importantly, growth recovery should not be equated with food-safety recovery. Future studies should therefore assess whether BR-induced biomass restoration is accompanied by lower particle accumulation, reduced co-contaminant transfer, and preserved nutritional quality in harvested organs. BRs should not be viewed as plastic degraders or substitutes for reducing plastic inputs. Their value lies in strengthening plant resilience where contamination already exists. By limiting NP translocation, restoring the AsA-GSH cycle, preserving PSII function, reprogramming hormone-metabolite networks, and stabilizing rhizosphere interactions, BRs offer a mechanistically plausible strategy to protect crops in plastic-impacted soils (Gao et al., 2023; Zhang et al., 2024; Gui et al., 2025; Zhang et al., 2025; Zhuang et al., 2025). The next challenge is to move from controlled proof-of-concept experiments to realistic soil systems in which BR-mediated resilience can be evaluated in the context of productivity, food safety, and long-term ecosystem health.

**Table 1:** Expanded direct and supporting evidence for brassinosteroid-mediated mitigation of micro- and nanoplastic stress in plants.

Evidence type	Plant/system	Plastic particle details	Plastic dose and exposure	BR treatment: compound, dose, application	Key observations	Mechanistic interpretation and cautions	Reference
Direct mitigation; edible-plant NP uptake and seedling phytotoxicity	Tomato (*Solanum lycopersicum*) seedlings and fruit; preliminary PS-NP accumulation screen across ten edible plant species.	Polystyrene nanoplastics (PS-NPs), non-surface-modified; nominal about 60 nm. In Hoagland solution: about 63.69 nm for non-fluorescent PS-NPs and 95.07 nm for fluorescent PS-NPs; zeta potential about -25.3 and -28.3 mV, respectively. PS-NPs accumulated mainly in xylem regions of edible tissues.	Main tomato assay: 50 mg L^-1^ PS-NPs for 14 d. Toxicity screen also included 10, 50, and 100 mg L^-1^. Aquaporin inhibition assay: 50 mg L^-1^ PS-NPs plus 0.05 mM AgNO_3_ for 7 d.	Reported as exogenous brassinosteroids (BRs); the available article text does not identify the analogue as BL or 24-epiBL; 10, 50, and 250 nM were screened; 50 nM was selected because it reduced PS-NP accumulation, whereas 250 nM suppressed tomato growth. Treatment was supplied exogenously in the culture system, not as a foliar spray in the described seedling assay.	50 nM BRs reduced PS-NP fluorescence in tomato fruit to near-control levels; mitigated PS-NP-induced reductions in fresh weight and plant height; decreased H_2_O_2_ and MDA; lowered stress-elevated SOD, POD, and CAT activities relative to PS-NP exposure alone.	BRs appear to reduce NP accumulation through aquaporin-associated transport control and redox stabilization. PS-NPs upregulated TIP2-1, TIP2-2, PIP2-6, PIP2-8, SIP2-1, and NIP1-2, whereas BRs downregulated TIP2-1, TIP2-2, PIP2-6, PIP2-8, PIP2-9, SIP2-1, and NIP1-2. Interpret aquaporins as transport/hydraulic regulators, not necessarily as pores through which intact NPs pass.	Gao et al. (2023)
Direct mitigation; medicinal plant physiology and secondary metabolism	*Pinellia ternata* pot experiment; leaves, tubers, photosynthesis, ROS metabolism, AsA-GSH cycle, and secondary metabolites were measured.	Polyethylene microplastics (PE-MPs), 13 µm particles/granules used as an agricultural PE model.	Soil amendment with 1% PE-MPs (w/w). PE-MPs and air-dried soil were mixed evenly and stabilized for 10 d before transplanting. Experimental design: MP 0/1% x BR 0/0.1 mg L^-1^.	0.1 mg L^-1^ BL (reported as BR in the paper) foliar spray for 7 d after the three leaves were fully unfolded; samples were collected 30 d after spraying.	MP reduced plant height (15.0%), Fv/Fm (3.2%), SOD (15.0%), and APX (5.1%), but increased O_2_^.-^ production (29.6%), GSH (4.4%), flavonoids (6.8%), alkaloids (75%), and β-sitosterol (26.5%). Under MP stress, BL increased plant height (15.7%), aboveground biomass (16.1%), underground biomass (10.3%), carotenoids (11.8%), GSH (4.2%), Fv/Fm (2.9%), SOD (32.2%), GR (21.08%), and MDHAR (20.9%).	BL functions as a rescue regulator rather than a plastic detoxifier. It improves PSII photochemistry, carotenoid-linked photoprotection, redox homeostasis, and AsA-GSH cycling, while moderating stress-related carbon allocation into secondary metabolism.	Zhang et al. (2024)
Direct mitigation; germination and early seedling growth	Rice (*Oryza sativa*, cultivar Zhe Fu No. 7) seed germination in Petri dishes for 7 d at 25 °C.	Polystyrene particles: micro-sized PS microspheres (50 µm MPs) and nano-sized PS microspheres (50 nm NPs). Stock suspension contained 2.5% solids; working solutions were sonicated for 30 min.	MP/NP treatments used 0, 200, 500, 1000, and 1500 mg L^-1^ in the germination assay; the combined BR plus plastic test used the high PS dose of 1500 mg L^-1^. Each dish contained 20 seeds and 5 mL treatment solution; three replicates.	0.01, 0.10, and 1.00 mg L^-1^ 24-epibrassinolide (24-epiBL), combined treatment used 0.01 mg L^-1^ 24-epiBL plus 1500 mg L^-1^ MPs or NPs.	NPs were more inhibitory than MPs. At 1500 mg L^-1^, NPs reduced root length, shoot length, fresh weight, dry weight, and vigor index by about 26%, 14%, 18%, 18%, and 22%, respectively. Adding 0.01 mg L^-1^ 24-epiBL to NPs increased root length, shoot length, fresh weight, dry weight, and vigor index by 43%, 27%, 20%, 13%, and 44% relative to NPs alone; BR+MPs also improved biomass indices.	24-epiBL protects early development mainly by redox regulation. BR+MP/NP treatments reduced POD, CAT, and MDA toward control levels and normalized SOD activity. The response is consistent with BR-mediated antioxidant priming and ROS homeostasis, not plastic degradation.	Gui et al. (2025)
Direct plant-soil interface evidence; rhizosphere microecology	*Pinellia ternata* rhizosphere/pot experiment; root morphology, soil physicochemical properties, enzymes, and bacterial/fungal communities were assessed.	Polyethylene microplastics (PE-MPs), 13 µm; SEM showed fragments, fibers, and irregular particles with rough surfaces and cracks.	1% PE-MPs (w/w) mixed with dried soil and stored/equilibrated for 10 d before planting. Plants were harvested on day 30 after transplanting.	0.1 mg L^-1^ brassinolide (BL; reported as BR) foliar spraying for one week after full leaf unfolding. Soil was covered during spraying to prevent direct BR addition to the soil.	BR+MP reduced root length (35.8% vs control) but maintained/increased root diameter, suggesting architectural reprogramming. Under MP stress, BL decreased soil CAT activity and increased urease activity; BL partially mitigated microbial dysbiosis by reducing pathogenic Rhizoctonia and improving the relative abundance of beneficial or less detrimental fungal groups compared with MP alone.	BL effects extend from plant tissues to the rhizosphere, probably through altered root architecture, exudation, nutrient demand, and stress signaling. The row should be framed as rhizosphere stabilization, not simple root-growth promotion, because root elongation decreased while radial/root-soil traits shifted.	Zhang et al. (2025)
Supporting evidence; hormone-network context, no exogenous BR rescue	Cucumber (*Cucumis sativus*, Jinyou No. 1); hydroponic and soil systems combined with transcriptomics and metabolomics.	Hydroponics: fluorescent PS microspheres of 0.1 µm and 5 µm. Soil: PVC, PE, and PS microplastics sieved to <13 µm; DOP used as a representative phthalate plasticizer.	Hydroponics: 10 mg L^-1^ PS. Soil: 0.05 g kg^-1^ PVC, PE, or PS; PS+DOP treatment combined 0.05 g kg^-1^ PS with 10 mg kg^-1^ DOP. Plants were cultivated for 74 d; seven replicates per treatment.	No exogenous BR applied. Endogenous BR-related regulation was inferred from BR biosynthesis/homeostasis genes, including DET2, CYP92A6, and CYP734A1. Metabolomics showed no significant accumulation of BRs, suggesting homeostatic compensation.	Microplastics and DOP primarily affected tryptophan-dependent auxin biosynthesis, CK *de novo* synthesis, GA metabolism, ABA-related ABA2 expression, ethylene biosynthesis, JA accumulation, and BR homeostasis. Smaller 0.1 µm PS was more readily absorbed/translocated than larger PS and was associated with reduced IAA biosynthesis efficiency. PE and PS affected hormone biosynthesis more strongly than PVC; PS+DOP synergistically enhanced JA.	This study supports the review concept that plastic stress rewires hormone networks, including endogenous BR homeostasis, and therefore provides rationale for BR-centered crosstalk experiments. It should not be cited as direct evidence that exogenous BR mitigates MP/NP stress.	Zhuang et al. (2025)

24-epiBL, 24-epibrassinolide; ABA, abscisic acid; AgNO_3_, silver nitrate; APX, ascorbate peroxidase; AsA-GSH, ascorbate-glutathione; BL, brassinolide; BR, brassinosteroid; CAT, catalase; CK, cytokinin; DOP, di-*n*-octyl phthalate; GA, gibberellin; GR, glutathione reductase; GSH, reduced glutathione; H_2_O_2_, hydrogen peroxide; IAA, indole-3-acetic acid; JA, jasmonic acid; MDA, malondialdehyde; MDHAR, monodehydroascorbate reductase; MP, microplastic; NP, nanoplastic; PE, polyethylene; POD, peroxidase; PS, polystyrene; PSII, photosystem II; PVC, polyvinyl chloride; ROS, reactive oxygen species; SOD, superoxide dismutase; Fv/Fm, maximum quantum efficiency of PSII.
